# Change in Proteolytic Profile in Heifers After Oligofructose Overload

**DOI:** 10.3389/fvets.2020.580375

**Published:** 2020-12-18

**Authors:** Shuaichen Li, Xiaoyan Zheng, Mengyuan Ding, Ze Tao, Jiantao Zhang, Na Zhang

**Affiliations:** ^1^College of Veterinary Medicine, Northeast Agricultural University, Harbin, China; ^2^Heilongjiang Key Laboratory for Laboratory Animals and Comparative Medicine, Northeast Agricultural University, Harbin, China; ^3^The Key Laboratory of Dairy Science of Education Ministry, Northeast Agricultural University, Harbin, China

**Keywords:** bovine, lameness, laminitis, oligofructose, protease

## Abstract

Laminitis in cattle is an important underlying cause of lameness, which leads to a significant reduction in economic and animal welfare. Nevertheless, the disordered pathological processes of laminitis remain unclear. Several proteinases are probably involved in the disorder of basement membrane (BM) metabolism in laminitis, for instance, matrix metalloproteinases (MMPs), neutrophil elastase (NE), and myeloperoxidase (MPO). This study aimed to investigate the change in proteolytic profile in circulating and lamellar tissues using an oligofructose (OF) overload-induced laminitis model in heifers. Twelve clinically healthy and nonlame Chinese Holstein heifers were recruited and randomly divided into two groups: OF-induced and control (CON). The OF-induced heifers group (*n* = 6) was administered 17 g/kg of body weight (BW) of OF dissolved in 2 L/100 kg of BW of tap water via the oral-rumen tube. The CON group (*n* = 6) was given an equal volume of tap water. The plasma samples were collected 0, 6, 12, 18, 24, 36, 48, 60, and 72 h after administration, and the lamellar samples were collected 72 h after euthanasia. The plasma samples were analyzed by zymography and reverse zymography. Histological examination, zymography, reverse zymography, and Western blot of lamellar samples were conducted. In the plasma of the OF-induced group, the pro-MMP9 activity increased from 36 h (*P* < 0.001) to 60 h (*P* < 0.05). Moreover, the plasma tissue inhibitors of metalloproteinase 1 (TIMP1) activity decreased after 18 h (*P* < 0.05), while the ratio of pro-MMP9 to TIMP1 and TIMP2 increased after 18 h (*P* < 0.001) and 48 h (*P* < 0.05), respectively. The act-MMP2, pro-MMP9, and act-MMP9 activities increased in the lamellar tissue of the OF-induced group compared with the CON group (*P* < 0.05). In addition, the expression of lamellar NE protein was higher in the OF-induced group (*P* < 0.01), while no change was found in lamellar MPO protein compared with the CON group. In conclusion, increased pro-MMP9 combined with decreased TIMP1 activity in the circulation might have caused the activation of blood neutrophils, while the activation of proteolytic enzymes in lamellar tissue probably led to the dysfunction of BM in the OF-induced group.

## Introduction

Laminitis in cattle is defined as a systemic disease with local manifestation in the claws. Many diseases of the claw capsule are supposed to be associated with laminitis, such as sole ulcer and white line disease (1). However, the pathogenesis of laminitis has not been completely elucidated because its early clinical symptoms are usually ephemeral or imperceptible. Previous studies reported that drenching oligofructose (OF) in heifers led to the signs of lameness and claw pain within 72 h (2), which mimicked the model of acute laminitis in horses. The degradation of the basement membrane (BM) in lamellar tissue was an important histological change in acute laminitis both cattle and horses after OF overload (3, 4). Several proteinases were probably involved in the disorder of BM metabolism, for instance, matrix metalloproteinases (MMPs), neutrophil elastase (NE), and myeloperoxidase (MPO).

MMPs are a family of Ca^2+^ dependent and Zn^2+^-containing endopeptidases that degrade extracellular matrix (ECM) and connective tissue proteins (5). Currently, among these MMPs, much attention has been focused on the role of MMP2 (gelatinase A) and MMP9 (gelatinase B) in laminitis (6). MMP2 is constitutively produced by many cell types, but MMP9 is mainly expressed by leukocytes, fibroblasts and keratinocytes. In normal tissues, MMP activity is inhibited by endogenous tissue inhibitors of metalloproteinases (TIMPs). TIMP1 binds specifically with pro-MMP9 and TIMP2 with pro-MMP2, while the ultimate effect on ECM turnover depends on the MMP/TIMP ratio (1). Both the activity and quantitation of MMP2 and MMP9 increased in the ulcerated bovine claw tissue, while diseased tissue at ulcer sites had lower TIMP2 expression in a previous study (7). In OF-induced equine laminitis, the plasma MMP9 activity was elevated from 16 h to 48 h and the pro-MMP2, pro-MMP9, and MMP9 activities increased in the lamellar tissue (6, 8). Furthermore, the expression of *TIMP2* gene significantly decreased in the lamellar tissue of equine with OF- or starch-induced laminitis (6, 9).

NE and MPO are mainly produced by neutrophils. NE has been recognized as one of the most destructive enzymes as it can degrade almost all ECM and key plasma proteins (10). MPO can transform H_2_O_2_ into hypochlorous acid and kill pathogenic invaders but to cause cytotoxic damage to the host tissue (11). In bovine medicine, several studies have reported on the effect of NE or MPO on intestinal parasitism or mastitis (12, 13), while their effect on other disease has been underreported. Lamellar NE protein was found to increase in horses with black walnut extract (BWE)-induced laminitis and chronic laminitis (14, 15). Similarly, lamellar MPO concentrations increased in BWE-induced laminitis and strongly correlated with the pro-MMP9 activity in horses with starch-induced laminitis (15, 16). These increased activities of proteinases probably caused the degradation of lamellar matrix components. However, the role of these proteinases was unclear in OF induced bovine laminitis. Previously, we demonstrated the activation of blood neutrophil and the inflammation reaction of lamellar tissue after OF overload (17, 18). This study aimed to investigate the changes in the proteolytic profile in plasma and lamellar tissue in heifers with OF-induced laminitis.

## Materials and Methods

### Ethics Statement

All procedures, treatments, and animal care were conducted under the approval of Institutional Animal Care and Use Committee of Northeast Agricultural University (approved protocol number SRM-13) in accordance with the Laboratory Animal Guideline for ethical review of animal welfare.

### Animals

Twelve Chinese Holstein heifers (aged 18–26 months and weighing 335–403 kg) were used in this study, which were clinically healthy with normal locomotion and without claw lesions. All heifers were housed in tie stalls with concrete floor for 30 days of acclimation. During this period, the heifers were fed with mixed grass and lucerne hay *ad libitum* and had free access to water.

### Experimental Design and Treatments

The experimental animals were randomly divided into two groups: OF-induced and control (**CON**). In the OF-induced group (*n* = 6), 17 g/kg of BW of OF dissolved in 2 L/100 kg of BW tap water was given via an oral-rumen tube (length 2.2 m, diameter 25 mm) at 0 h. In the CON group (*n* = 6), tap water at the same amount was given. Three days before the primary overload, 5% of the primary dose was provided to all heifers. For animal welfare, supportive therapy was provided in the form of calcium borogluconate after 18 h, and Ringer's solution (Heping Animal Medicine Co., Ltd, Harbin, China) and sodium bicarbonate 18 and 24 h after OF overload, as described in previous studies (17). In addition, Locomotion assessment was performed at each time point according to Sprecher et al. (19).

### Plasma and Lamellar Tissue Collection

Twenty mls of blood sample of each heifer was collected from jugular vein into sodium heparin evacuated tubes 0, 6, 12, 18, 24, 36, 48, 60, and 72 h after administration. The tubes were centrifuged for 15 min at 3,500 rpm within 1 h after collection. The plasma was transferred and frozen at −80°C.

All heifers were euthanized, stunning with a captive bolt then exsanguination by severing jugular vein and trachea, after 72 h of overload. The front claws were removed from the metacarpophalangeal joint using a bone cutter. The medial and lateral claws were separated and cut into small pieces (~1 cm^2^) containing horn, lamellar tissue and bone, as describe by Danscher et al. (20). Several tissue blocks were fixed in 10% neutral buffered formalin for histological examination. The hoof wall and bones were removed in others using a scalpel on the ice, immediately frozen in liquid nitrogen for several seconds, and subsequently stored at −80°C for zymography and Western blot analysis.

### Histological Examination

Tissue samples for histological examination were separated from the horn and the bone after 24-h fixation. These samples were processed through a graded series of ethanol and xylene for dehydration and then embedded in paraffin. Tissue sections (5 μm thick) were cut and stained with periodic acid-Schiff (PAS). More histological section and description were described elsewhere (18).

### Zymography and Reverse Zymography

Plasma and lamellar tissue samples were used for measuring the MMP and TIMP activities with modifications according to previously described gelatin zymography (5, 21) and reverse zymography (22, 23), respectively.

For plasma, 50 μL of the sample was diluted to 1:20 with running buffer (50 mM Tris/HCl, 200 mM glycine, and 0.1% SDS). For lamellar tissue, 100 mg of the sample was homogenized using RIPA lysis buffer (P0013K, Beyotime Biotechnology, China; 1% Triton X-100, 1% deoxycholate, 0.1% SDS, without protease inhibitor). The samples of plasma and lamellar tissue were adjusted for protein concentration using BCA protein assay and then mixed with an equal volume of a sample buffer (50 mM Tris/HCl, 20% glycerol, 4% SDS, and 0.005% bromophenol blue).

Equal amounts of protein samples (20 μg) were loaded onto 8% polyacrylamide gel containing 1% bovine gelatin to quantify MMPs or 12% polyacrylamide gel containing 1% bovine gelatin and 0.1 μg/mL recombinant human MMP 2 (C377, Novoprotein Technology, China) to quantify TIMPs. After electrophoresis for 90 min with 125 V at 4°C, the gels were washed twice in a renaturing buffer (2.5% Triton X-100 in deionized water, pH 7.6), and then incubated for 18 h at 37°C in developing buffer (50 mM Tris/HCl, 0.2 M NaCl, 5 mM CaCl_2_, 0.02% Brij35, and 1 μM ZnCl_2_, pH 7.6). Subsequently, the gels were stained with 0.25% Coomassie blue R-250 and destained in a solution of methanol, acetic acid and distilled water (4.5:1:4.5). After destained, the gels showed clear bands on a blue background of undegraded gelatin in zymography or dark bands against the background where gelatin was degraded in reverse zymography. The gels were imaged using the Gel 1000 imager system (Sage Creation Science, China). The pre-stained protein ladder (26616, Thermo Fisher Scientific, USA) was used for the estimated molecular weight of the band, and the band intensity was measured using ImageJ software.

### Western Blot Analysis

The samples of lamellar tissue were homogenized using RIPA lysis buffer (P0013B, Beyotime Biotechnology, China), and total protein was quantified using the BCA method and boiled for 10 min. Aliquots of protein samples (25 μg) were separated on 10% SDS-PAGE gels until bromophenol blue dye reached the bottom of the gel and then transferred to nitrocellulose membranes. The membranes were blocked in 5% skimmed milk for 2 h at room temperature and then incubated with the diluted primary antibody overnight at 4°C. The primary antibodies were as follows: NE (1:200, SC-55548) and MPO (1:500, SC-52707) from Santa Cruz Biotechnology (USA) and β-actin (1:2000, D110001) from BBI Life Sciences (China). After washing with TBS-T, the membranes were incubated for 2 h at room temperature with a diluted secondary antibody. The secondary antibodies m-IgGκ BP-HRP (1:1000, SC-516102) and HRP-conjugated goat anti-rabbit IgG (1:5000, D110058) were procured from Santa Cruz Biotechnology and BBI Life Sciences, respectively. The protein bands were visualized using ECL Western blot detection reagent and quantified by densitometry using ImageJ software.

### Statistical Analysis

Statistical analysis was performed using GraphPad Prism version 7.04 (GraphPad Software Inc., CA, USA). All data were presented as means ± standard error. The Shapiro-Wilk normality test was used to determine normal distribution. Either two-way repeated measure ANOVA and Bonferroni's multiple comparisons test was conducted for plasma sample or *t*-tests (Mann-Whitney test for parametric data) were used for lamellar tissue. For all comparisons, a value of *P* < 0.05 was considered significant.

## Results

### Clinical Signs

All heifers in the OF-induced group exhibited lameness as expressed by >2 locomotion scores after 72 h (19). In addition, distinct symptoms of acute ruminal and systemic acidosis were observed in all heifers in this group, including depression, loss of appetence, watery diarrhea, and transient fever, consistent with a previous study (17).

### Histological Examination

**Figure 1:**
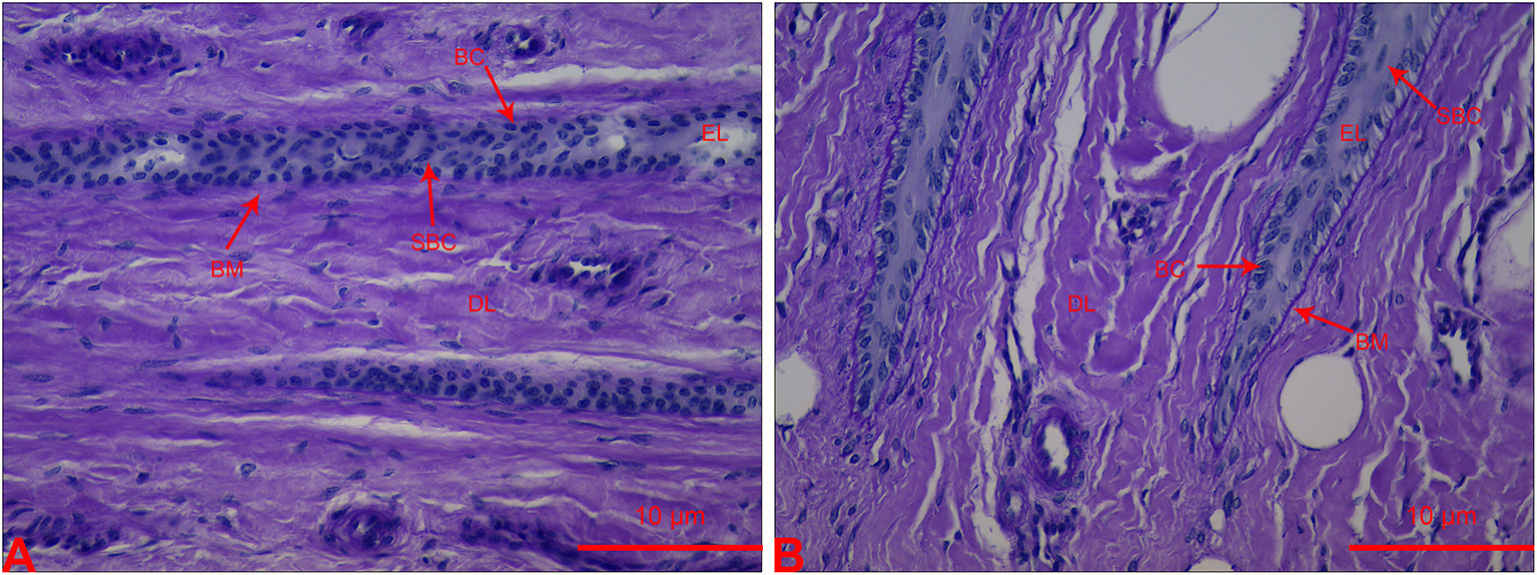
Section of lamellar layer obtained from heifers in the OF-induced group **(A)** and CON group **(B)** stained with periodic acid-Schiff. BC, basal cells; BM, basement membrane; DL, dermal lamellae; EL, epidermal lamellae; SBC, suprabasal cells.

The lamellar sections from heifers in the CON group had an orderly distribution of basal cells (**BC**) and continuous structure of BM. A few suprabasal cells (**SBC**) were present in the epidermis layer (**EL**). Most BC and SBC showed cylindrical cell morphology (Figure 1B). However, the lamellar section of heifers in the OF-induced group showed that BC and SBC were randomly distributed in EL. The BM had a light-stained and attenuated appearance. Moreover, BC and SBC were round in shape (Figure 1).

### MMP and TIMP Activities in Plasma

**Figure 2:**
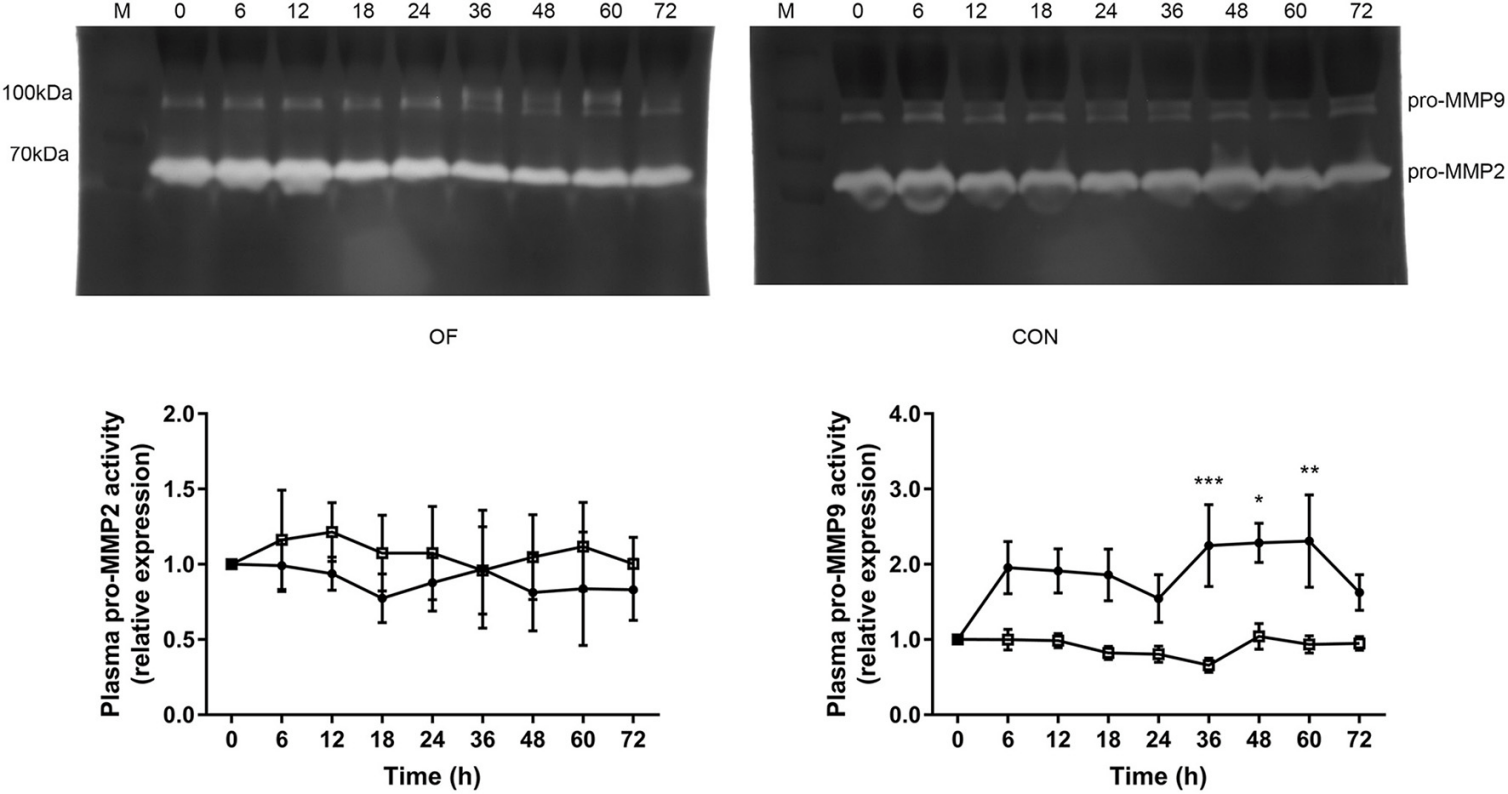
Changes in the MMP activities in plasma of heifers in the OF-induced group (*n* = 6) or CON group (*n* = 6). Representative gelatin zymography and densitometry analysis of pro-MMP2 and pro-MMP9 in plasma. The results were expressed relative to 0 h for each group of heifers and shown as means ± SEM. *P < 0.05, **P < 0.01, ***P < 0.01 (repeated measures ANOVA); M is protein molecular weight markers. **•** = OF, **□** = CON.

In plasma zymography, the pro-MMP9 activity significantly increased from 36 h (*P* < 0.001) to 60 h (*P* < 0.05), while no difference was observed in pro-MMP2 in the OF-induced group. In the CON group, no changes were found in the pro-MMP2 and pro-MMP9 activities. In addition, the act-MMP2 and act-MMP9 activities in plasma were too low to determine in both CON and OF-induced group (Figure 2).

**Figure 3:**
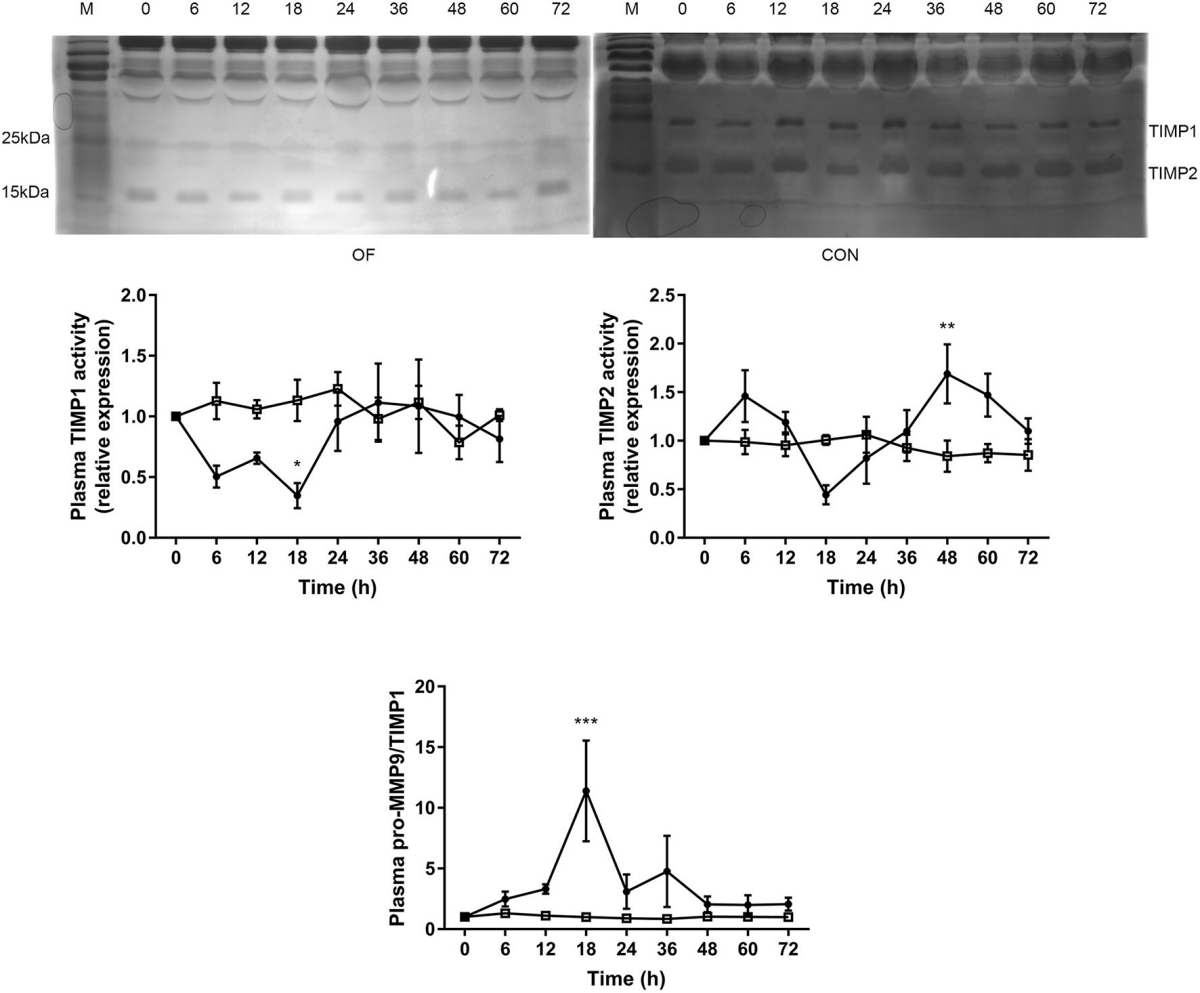
Changes in the TIMP activities in plasma of heifers in the OF-induced group (*n* = 6) or CON group (*n* = 6). Representative reverse zymography and densitometry analysis of TIMP1 and TIMP2 in plasma and the pro-MMP9/TIMP1 ratio in plasma. The results were expressed relative to 0 h for each group of heifers and shown as means ± SEM. *P < 0.05, **P < 0.01, ***P < 0.01 (repeated measures ANOVA); M is protein molecular weight markers. **•** = OF, **□** = CON.

The TIMP1 activity was significantly lower after 18 h (*P* < 0.05), while the TIMP2 activity was significantly increased after 48 h in the OF-induced group compared with the CON group (*P* < 0.01). Furthermore, dramatically increased pro-MMP9 to TIMP1 ratio was observed after 18 h in the OF-induced group (*P* < 0.001; Figure 3).

### MMP and TIMP Activities in Lamellar Tissue

**Figure 4:**
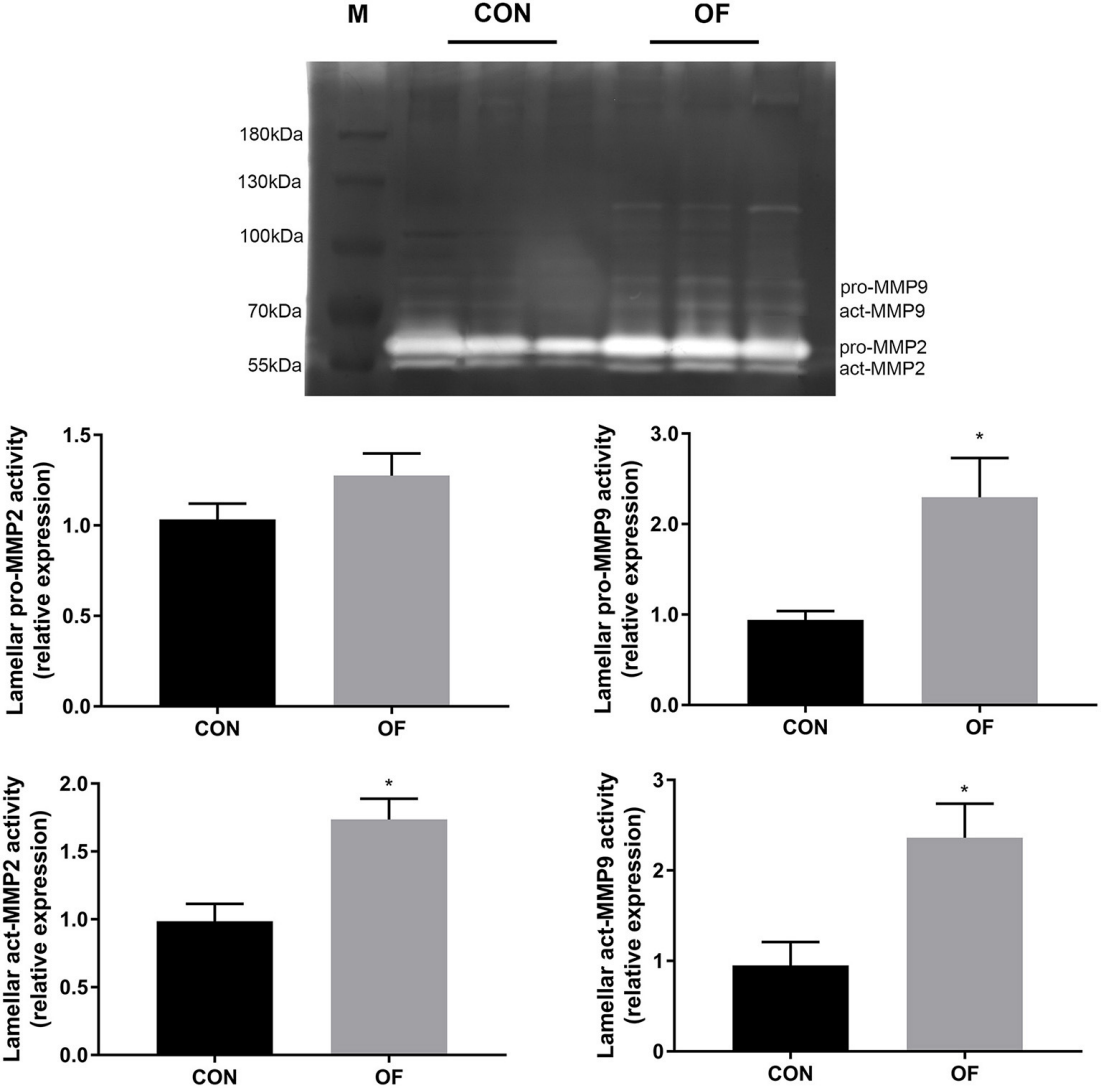
Changes of MMP activities in lamellar tissue of heifers in the OF-induced group (*n* = 6) or CON group (*n* = 6). Representative gelatin zymography and densitometry analysis of pro-MMP2 and pro-MMP9 in lamellar tissue. The results were expressed relative to the CON group and shown as means ± SEM. *P < 0.05 (unpaired *t* test). M is protein molecular weight markers. Black bars represent the CON group and gray bars represent the OF-induced group.

In lamellar zymography, the act-MMP2, pro-MMP9, and act-MMP9 activities were greater in the OF-induced group than in the CON group (*P* < 0.05), while no differences were observed in pro-MMP2 activity (Figure 4).

**Figure 5:**
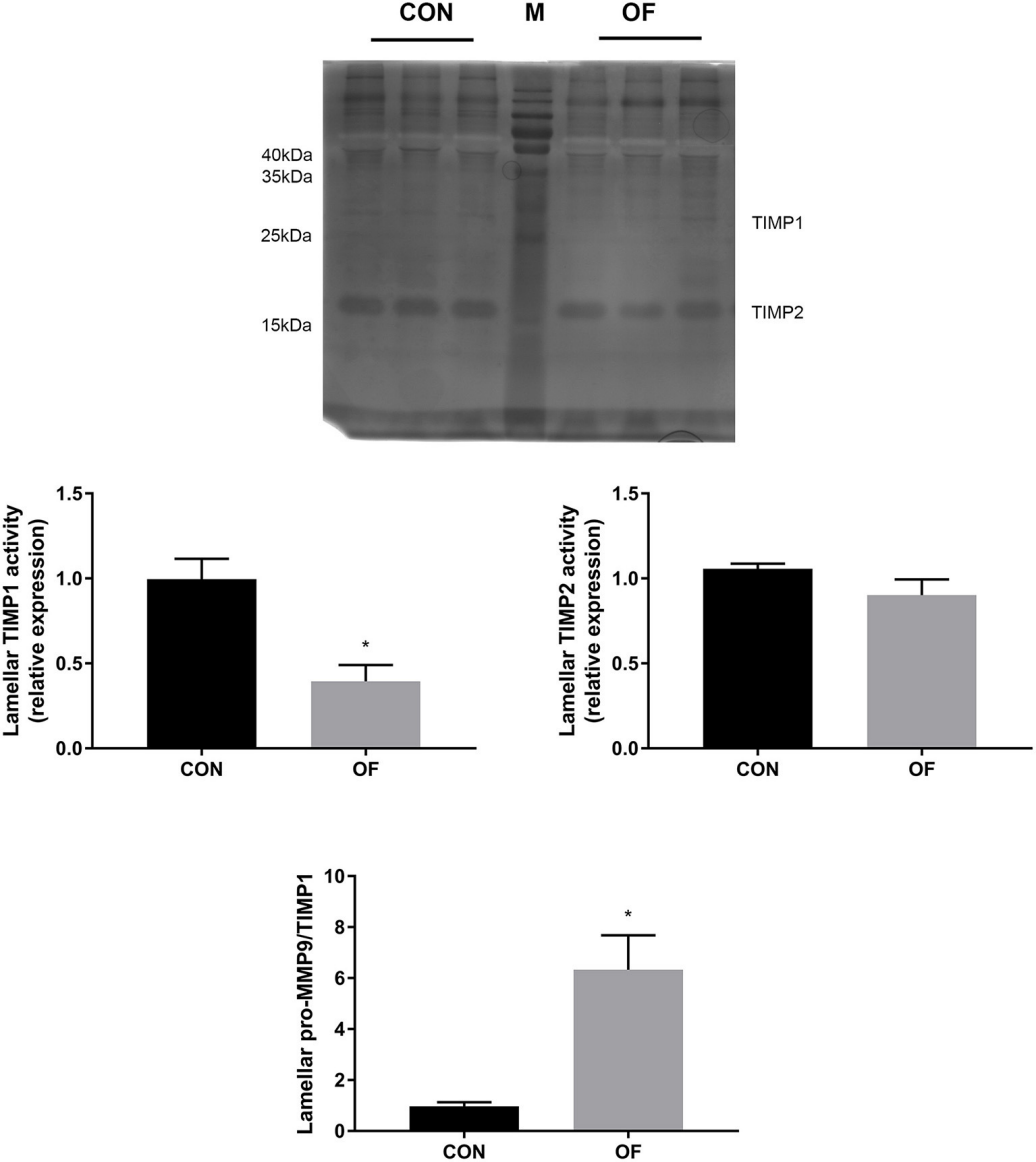
Changes of TIMP activities in lamellar tissue of heifers in the OF-induced group (*n* = 6) or CON group (*n* = 6). Representative reverse zymography and densitometry analysis of TIMP1 and TIMP2 in lamellar tissue and the pro-MMP9/TIMP1 ratio in lamellar tissue. The results were expressed relative to the CON group and shown as means ± SEM. *P < 0.05 (unpaired *t* test). M is protein molecular weight markers. Black bars represent the CON group and gray bars represent the OF-induced group.

The results of reverse zymography showed that the TIMP1 activity significantly decreased (*P* < 0.05) and TIMP 2 had no changes in the OF-induced group. In addition, an increase in the MMP9/TIMP1 ratio was observed in the OF-induced group (*P* < 0.05; Figure 5).

### Expression of NE and MPO in Lamellar Tissue

**Figure 6:**
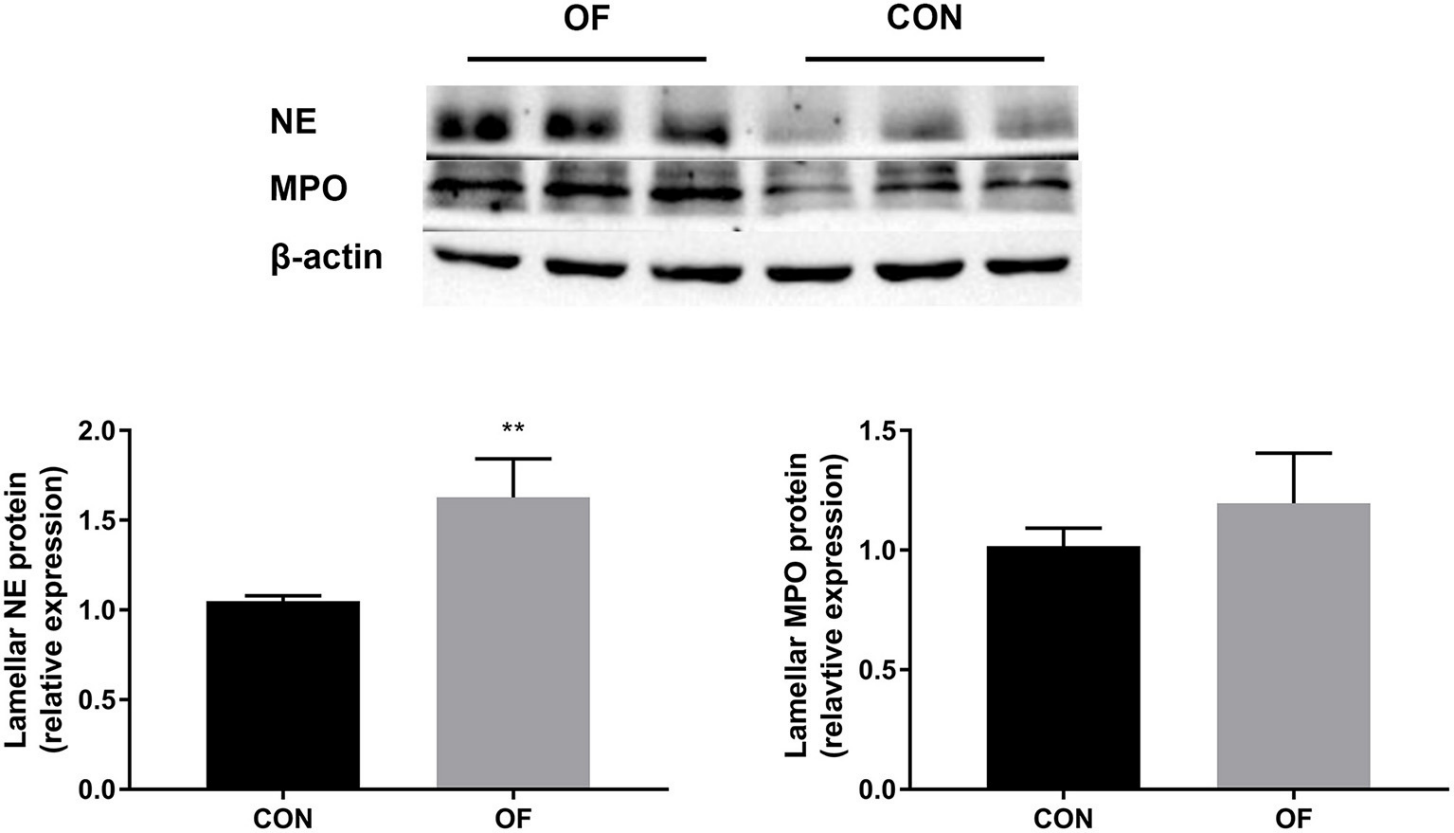
Changes in NE and MPO protein level in the lamellar tissue of heifers in the OF-induced group (*n* = 6) or CON group (*n* = 6). Representative Western blot and densitometry analyses of NE and MPO in lamellar tissue. The results were expressed relative to the CON group and shown as means ± SEM. **P < 0.01 (unpaired *t* test). Black bars represent the CON group and gray bars represent the OF-induced group.

The results of Western blot analysis showed a significant increase in NE protein in the OF-induced group (*P* < 0.01) compared with the CON group, whereas no change was observed in the expression of MPO protein (Figure 6).

## Discussion

OF-induced group showed increased locomotion scores and aberrant histological changes in lamellar tissue, which were similar with naturally and experimentally acquired laminitis in cattle (2, 24). It is suggested that bovine laminitis model successfully established in the present study that can be used in evaluating the change in proteolytic profile in plasma and lamellar tissue.

Several techniques have been used to determinate MMPs in various biological samples, for example enzyme linked immunosorbent assays (ELISA) and zymography. ELISA can provide quantitative data of MMPs, yet it is not sufficient to accurately describe protein activity *in vivo* (25). Therefore, the present study selects zymography for the analysis of MMP and TIMP in plasma and lamellar tissue. Zymography is based on separation of proteins by non-reducing SDS-PAGE gels which is embedded with specific substrate such as gelatin (5). During electrophoresis, sample are separated according to the molecular weight of the protein under denaturing (SDS) conditions. Both pro-enzyme and active forms of MMP are detected by the absence of gelatin in the gel, which are appear as white bands. Reverse zymography is a modified zymography to detect TIMP activity by adding MMP to gel before running, which is appear as dark blue bands (26).

The use of MMP and TIMP plasma levels as biomarkers in different clinical settings is increasing due to their diagnostic and prognostic value in human medicine (23, 27). Currently, only a few studies have been focused on plasma or milk levels of MMP (21, 28), while the plasma level of TIMPs in cattle is lack of relevant research. The present study showed that the pro-MMP9 activity increased in plasma from 36 to 60 h in the OF-induced group. Similarly, the plasma pro-MMP9 activity markedly increase in horse with OF-induced laminitis (8). Polymorphonuclear leukocytes are recognized as the major source of MMP9 in plasma (29). Increased plasma pro-MMP9 activity is consistent with previous research that showed the upregulated of *MMP9* mRNA in blood neutrophil during OF overload in heifers (17). In normal conditions, TIMP prevent excessive MMP activity, which bind non-covalently at a 1:1 ratio with high affinity to both pro- and active MMP catalytic sites (30). In the present study, the circulating TIMP1 activity and pro-MMP9/TIMP1 significantly decreased after 18 h. This generated an environment of higher proteolytic power, which may promote neutrophil recruitment and infiltration. Fugler et al. found that the use of MMP inhibitors in horses could decrease lipopolysaccharide-induced increases in plasma MMP activity (31). It was interesting that plasma TIMP2 activity was increased after 48 h, which might contribute to reduce the systemic symptoms in the late stage of induction.

BM is mostly made up of type IV collagen and laminin, and is frequently referred to as the dermoal-epidermal junction. It is also involved in regulating the proliferation and differentiation of mitotic cells in the living epidermis (1). MMP2 and MMP9 have been widely identified in association with the BM destruction and lamellar separation characteristic of acute laminitis in horses, while few studies have been conducted on bovine laminitis. In this study we have shown that increased activity of act-MMP2 and MMP9 seem to be responsible for the degration of lamellar BM after OF-induced laminitis in heifers, which is consistent well with OF-induced equine laminitis (6). In ulcerated bovine claw tissue with BM disruption, higher levels of MMPs (act-MMP2 and pro-MMP9) and lower levels of TIMP2 were observed in a previous study (7). Despite a marked increase in the neutrophil counts in blood and synovial fluid after OF overload in heifers (32, 33), only a few white blood cells were observed in lamellar tissue (20). Hence, the source of MMP9 in lamellar tissue need further confirmation. Furthermore, the transcription level of the lamellar *TIMP2* gene is reduced in horses with OF- or starch-induced laminitis, while the transcription level of *TIMP1* gene was randomly changed (6, 9). Unlike the aforementioned studies, lamellar TIMP1 decreased and the pro-MMP9/TIMP1 was markedly increased, although the lamellar pro-MMP2 and TIMP2 activities did not change in the present study. The balance of homeostasis is broken once too much MMP are produced and/or cytokines downregulate expression of TIMP (1). It has been demonstrated that pro-inflammatory cytokines (such as IL1 and TNF) in the hoof dermis and epidermis was increased in cattle with sole or hoof wall lesions (34). Furthermore, IL1 leads to the release of MMP9 and to the inhibition of TIMP1 in fibroblasts (35), which might be the cause of decreased lamellar TIMP1 activity. This study indicated that the change in MMP9 and TIMP1 plays a role in lamellar BM dysfunction after OF overload. Consequently, further studies are needed to confirm the alteration of MMP and TIMP in terms of gene transcription and protein quantitation.

Although MMP activity is likely to correlate with catalytic activity at BM, MMPs need to convert into the active form which depends on either oxidative (e.g., radical oxygen species) or proteolytic activity (e.g., NE) (14). Visser et al. found that MMPs appeared not to play an early role in the lamellar lesions after OF induced equine laminitis, and suggested that other proteases needed to be considered, such as NE and MPO (6). Thus, the present study evaluated the level of lamellar NE and MPO in heifers in the OF-induced and CON groups.

Both NE and MPO are primarily synthesized by neutrophils and then stored in azurophilic granules of neutrophils. They are generally considered to be a component and marker of inflammatory disorders. As in the case of horses with chronic laminitis, lamellar NE protein increased in heifers with OF-induced laminitis (15). Hidalgo et al. recorded the presence of neutrophil extracellular traps (NET) in synovial fluid after OF-induced (13 g/kg) acute ruminal acidosis by measuring cellular free DNA, while NE and MPO were components in NET (36). It is interesting that the level of MPO protein in lamellar tissue remained unchanged after OF overload, though MPO concentration in lamellar tissue had a strong positive correlation with pro-MMP9 activity in horse with laminitis (37). Lamellar MPO may be involved in the degradation of ECM proteins by nitration of constitutive collagen fibers and fibronectin in the development of equine laminitis (38). However, recent *in vitro* studies indicated that the lower MPO level might play a protective effect during lipopolysaccharide stimulated neutrophil in cow (39). Therefore, further investigation of MPO level in lamellar tissue is needed.

## Conclusions

The present study indicated that not only increased pro-MMP9 in plasma but also the higher level of act-MMP2, MMP9, and NE probably caused the degradation of lamellar separation in OF-induced laminitis in heifers. Together, the change in MMPs, TIMPs, and other proteases might be vital in the development of laminitis, which needs confirmation.

## Data Availability Statement

The original contributions presented in the study are included in the article/supplementary material, further inquiries can be directed to the corresponding author/s.

## Ethics Statement

The animal study was reviewed and approved by Institutional Animal Care and Use Committee of Northeast Agricultural University.

## Author Contributions

JZ, NZ, and SL conceived the animal experiments. SL, XZ, MD, and ZT performed experiment and analyzed data. SL wrote the manuscript. All authors contributed to the article and approved the submitted version.

## Conflict of Interest

The authors declare that the research was conducted in the absence of any commercial or financial relationships that could be construed as a potential conflict of interest.
